# A real-world study in advanced non-small cell lung cancer with de novo brain metastasis

**DOI:** 10.7150/jca.51411

**Published:** 2021-01-01

**Authors:** Lei Lei, Wen-xian Wang, Dong Wang, Li Lin, You-cai Zhu, Hong Wang, Li-ping Wang, Wu Zhuang, Mei-yu Fang, Bing Wan, Hui-jing Feng, Chun-wei Xu

**Affiliations:** 1 Department of Chemotherapy, Institute of Cancer Research and Basic Medical Sciences of Chinese Academy of Sciences, Cancer Hospital of University of Chinese Academy of Sciences, Zhejiang Cancer Hospital, Hangzhou Zhejiang 310022, People's Republic of China; 2 Department of Respiratory Medicine, Affiliated Jinling Hospital, Medical School of Nanjing University, Nanjing, Jiangsu 210002, People's Republic of China; 3 Department of Oncology, Peking University International Hospital, Beijing 102206, People's Republic of China; 4 Department of Thoracic Disease Center, Zhejiang Rongjun Hospital, Jiaxing Zhejiang 314000, People's Republic of China; 5 Department of Lung Cancer, The Fifth Medical Center, General Hospital of PLA, Beijing 100071, People's Republic of China; 6 Department of Thoracic Oncology, Baotou Cancer Hospital, Baotou Inner Mongolia 014000, People's Republic of China; 7 Department of Medical Oncology, Fujian Cancer Hospital, Fujian Medical University Cancer Hospital, Fuzhou Fujian 350014, People's Republic of China; 8 Department of Respiratory, The Affiliated Jiangning Hopsital of Nanjing Medical University, Nanjing Jiangsu 210002, People's Republic of China; 9 Department of Thoracic Oncology, Shanxi Academy of Medical Sciences, Shanxi Bethune Hospital, Taiyuan Shanxi 030032, People's Republic of China

**Keywords:** de novo brain metastasis, *EGFR*, EGFR-tyrosine kinase inhibitors (TKIs), non-small-cell lung cancer

## Abstract

Brain metastases are the major cause of life-expectancy shortened for patients with lung cancer. The prognostic value of *EGFR* mutation subtypes and survival benefit of EGFR-tyrosine kinase inhibitors (TKIs) in advanced non-small cell lung cancer (NSCLC) patients with de novo brain metastasis is still not clear. Here, we present a real-world study nation-wide focusing on the prognostic value of genomic and therapeutic factors in overall survival (OS) of those patients. We enrolled a total of 233 patients diagnosed with advanced NSCLC and de novo BM from multi-medical centers across China. The enrolled patients were divided into 4 groups, including *EGFR* 19del, *EGFR* L858R, *EGFR* wild-type, and *EGFR* unknown groups. The median OS of patients with* EGFR* mutations and all patients were 29.0 and 25.0 months, respectively. There was significant difference in OS of patients among *EGFR* 19del (n=76), *EGFR* L858R (n=94), *EGFR* wild-type (n=46) and *EGFR* unknown (n=17) groups (30.5* vs* 27.5 *vs* 16.0* vs* 25.0, P=0.025). Patients treated by icotinib showed better OS than gefitinib and erlotinib (31.0 *vs* 25.5 *vs* 26.5, P=0.02). There was a difference in OS of patients received the whole-brain radiotherapy (WBRT), stereotactic radiosurgery (SRS), or WBRT+SRS (20.0 *vs* 31.0 *vs* 30.0 months, P<0.001), respectively. In multivariate analysis, patients treated with icotinib had superior iPFS benefit than gefitinib and erlotinib (HR=0.86[95%CI (0.74-1.0)], P=0.04). Besides, the histology of non-adenocarcinomas, the number of BM (>3), and extracranial metastases status could have an independent negative impact on the OS of all patients (P<0.001). *EGFR* mutant NSCLC patients with de novo BM had a better OS than patients with *EGFR* wild type. Patients treated with icotinib had longer iPFS than gefitinib and erlotinib but not in OS. Non-adenocarcinomas, number of BM (>3) and extracranial metastases were independent negative prognostic factors in iPFS and OS of all patients. Prospective clinical trials are warranted to explore more effective multimodality in this population.

## Introduction

Brain metastases are the major cause of the poor prognosis and quality of life in all metastatic cancer patients 1, 2, especially in advanced non-small cell lung cancer (NSCLC) 3. About 10-35% of the NSCLC patients have de novo BM on the diagnosis of lung cancer, and more than 50% of patients would develop BM during the treatment course 4, 5. The conventional treatment options for advanced NSCLC patients with de novo BM include surgical resection, radiotherapy, and chemotherapy with a median overall survival (OS) that ranges from 4.0-31.0 months 6-8. Although the combination or sequential use of those therapies have been reported to improve the OS, the major hindrance for chemotherapy is still the barely penetrated blood-brain barrier (BBB) in terms of the big molecular weights of those drugs 9, 10.

The identification of oncogene-addicted NSCLC and the development of targeted therapy, like *EGFR* mutations and EGFR-TKIs, have launched the new era in advanced NSCLC 11. EGFR-TKIs have shown have remarkable intracranial activity and acceptable tolerance, and survival benefit in advanced NSCLC patients with BM 12. It has been shown that BM occurrence is more frequent in NSCLC patients with *EGFR* mutations 13, 14. Besides, the BM pattern and clinical outcome of NSCLC patients with BM could have an association with *EGFR* mutation subtypes 15-17. Of note, the efficacy of TKIs could also be heterogeneous in terms of evidence of low penetration rates through BBB among first- and second generations of TKIs 18, 19, but the increasing potential of penetration by combined therapy, such as radiotherapy 20.

However, most of the research has been conducted in NSCLC patients with metastatic BM in general or limited numbers of patients with de novo BM. The prognostic factors and survival benefit from updated therapies are worth to be discussed in this particular population who are treatment-naïve and longer survival could be expected 8, 21. In that case, we designed this real-world study to investigate the prognostic value of *EGFR* mutation subtypes and the efficacy of TKIs in NSCLC patients with de novo BM from multi-medical centers across China.

## Materials and methods

### Patient population

This study enrolled 233 patients diagnosed as NSCLC with de novo BM from multi-center hospitals in the southeast of China between October 2012 and October 2016. Inclusion criteria were: 1) all the diagnoses were pathologically confirmed on the primary or metastatic tumor using transthoracic needle biopsy or bronchoscopic biopsy, 2)* EGFR* mutations were detected from the primary or metastatic lesion of all patients, and 3) complete data of baseline clinicopathologic characteristics including age at diagnosis, gender, histology, number of intracranial metastasis, extracranial metastatic status, and ECOG performance status (PS). Exclusion criteria were: 1) patients with other malignant disease histories at the time of diagnosis (due to difficult calculations of recurrent events and double cancers may increase the risk of recurrence), 2) patients who received palliative surgery of primary tumor or metastatic BM were excluded (due to the small sample size and potential longer survival may confound the conclusion of this study).

All patients were grouped by the results of* EGFR* mutation testing, and patients with* EGFR* mutations took gefitinib, erlotinib, or icotinib as the first-line targeted therapy if they are asymptomatic BM at the initial diagnosis. Patients with symptomatic BM received radiotherapy as the first-line therapy and followed by systemic therapy (targeted or chemotherapy) according to the genomic and diagnostic characters. The regimens of chemotherapy were classified into pemetrexed-based and non-pemetrexed-based groups. Disease progression was determined based on the radiographic evidence according to Response Evaluation Criteria in Solid Tumors (RECIST) version 1.1. This retrospective study was approved by the ethical committee. All patients´ follow-up information was obtained from their last clinical visits, follow-up registration records, and follow-up phone records. The study was approved by Shanxi Academy of Medical Sciences, Shanxi Bethune Hospital Ethics Committee (NO.: YXLL-2015-004) and written informed consent was taken from all the patients.

### Study design

We have collected the clinicopathologic characteristics and survival information from all patients and compared patients in different groups of *EGFR* mutation subtypes, including* EGFR* 19del, *EGFR* L858R, *EGFR* wild-type, and *EGFR* unknown groups. The clinicopathologic characteristics of patients were categorized by age (<65, ≥65 years), gender (male, female), ECOG PS (0-1, 2-3), smoking status (never-smoker, smoker), primary tumor histology (non-adenocarcinoma, adenocarcinoma), number of BMs (limited, diffuse), extracranial metastases (yes or no), radiotherapy (WBRT, SRS, or combination of WBRT+SRS), EGFR-TKIs (gefitinib, erlotinib, icotinib), chemotherapy (non-pemetrexed-based, pemetrexed-based).

For the survival analysis, we mainly focus on comparing the correlation with* EGFR* mutation subtypes and EGFR-TKIs with the iPFS and OS of all patients, as well as other prognostic factors. The iPFS was defined as the duration of time from initial first-line treatment to intracranial progression. The OS was defined as the duration of time from the initial diagnosis until death or the most recent follow-up.

### Statistical Analysis

Chi-square test was used to test the association between the *EGFR* mutation subgroups and clinical categorical variables in all patients. Kaplan-Meier survival analysis and the log-rank test were used to compare the differences in iPFS and OS among patients with different characteristics. These survival comparisons were stratified by* EGFR* mutation status separately. Multivariate Cox regression was used to determine the independent prognostic factors for iPFS and OS of all patients adjusted by *EGFR* mutation subtypes, smoking status, primary tumor histology, number of BMs, extracranial metastases, radiotherapy and EGFR-TKIs. All statistical analyses were performed using R v.3.4.1.

## Results

### Baseline characteristics of patients

A total of 233 advanced NSCLC patients who were diagnosed with de novo BMs enrolled in the study. The median age of these patients was 56 years (23-84 years), more female than male patients (173* vs* 60), more never-smokers than smokers (161 *vs* 72) and 212 patients (91%) had *EGFR* PS of 0-1. These cases included 212 (91%) adenocarcinoma and 21(9%) non-adenocarcinoma cases. The *EGFR* 19 del mutation occurred in 32.6 (76%) patients, 40.4 (94%) patients had the *EGFR* exon 21 L858R mutation, 46 (19.7%) *EGFR* wild-type and 17 (7.3%) *EGFR* unknown status. A total of 105 (45.1%) patients received icotinib (125 mg/3 times daily), 46 (19.7%) patients gefitinib (250 mg/d) and 36 (15.5%) erlotinib (150 mg/d). More than half of patients had extracranial metastasis (134/233, 57.5%). Those patients who received upfront WBRT (30Gy/10f/2W), SRS or WBRT+SRS were 113 (52.1%), 30 (13.8%) and 5 (2.3%), respectively. A total of 70 patients (30.5%) were treated with pemetrexed-based chemotherapy (pemetrexed 500 mg/m^2^ on day 1, every 3ws) as the first-line treatment, while 162 (69.5%) received non-pemetrexed-based chemotherapy instead. The patient characteristics at baseline are detailed in **Table 1**.

### Survival outcomes

**Figure 1:**
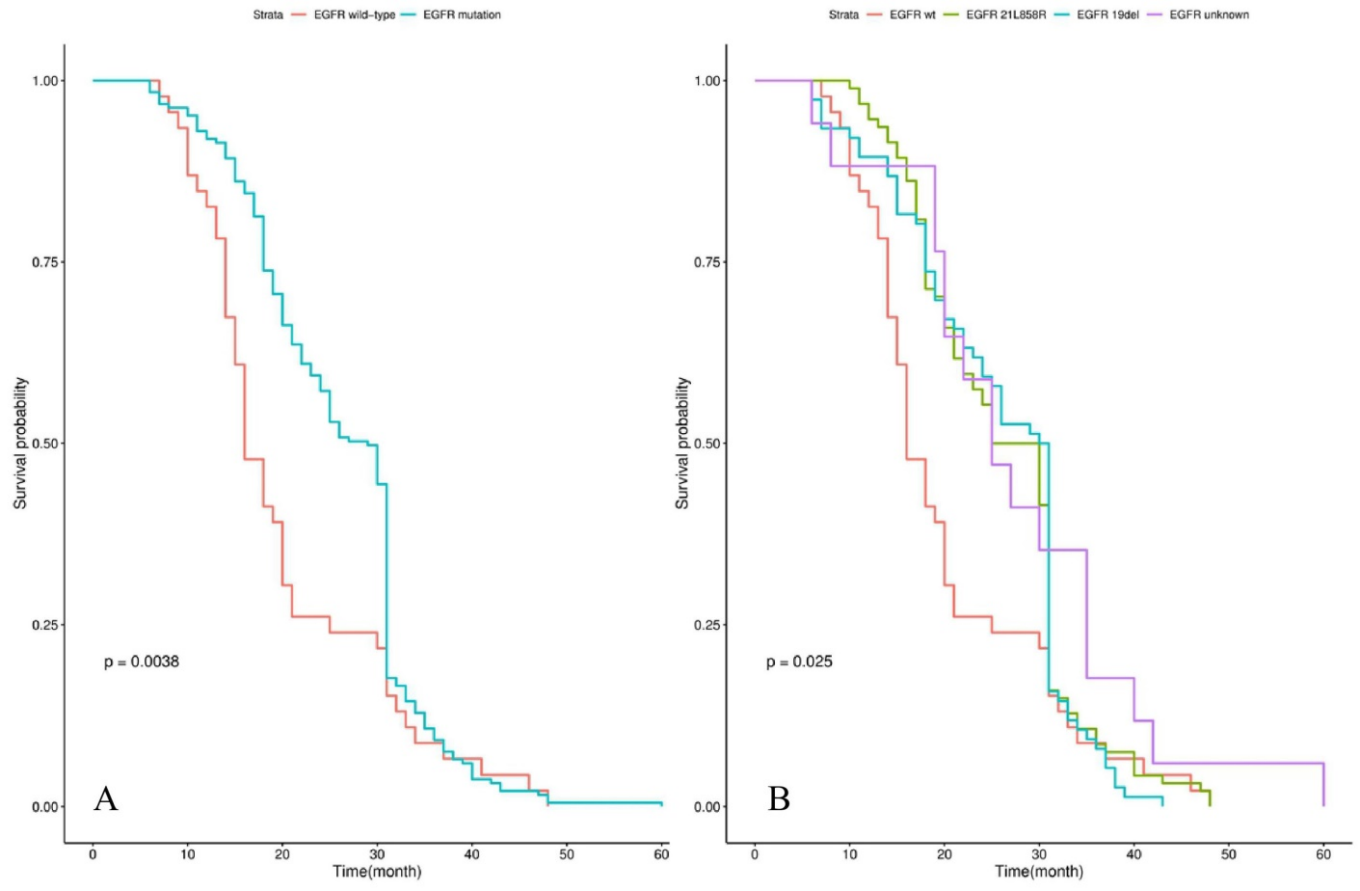
Kaplan-Meier curve analyses of OS in all NSCLC patients with de novo brain metastases. Kaplan-Meier curve was stratified by EGFR mutation status (EGFR wild-type vs EGFR mutation) in A; EGFR mutation types (exon 21 L858R *vs* exon 19 deletion *vs* unknown mutation) in B. *Abbreviations: OS, overall survival; NSCLC, non-small cell lung cancer; EGFR, epidermal growth factor receptor.*

**Figure 2:**
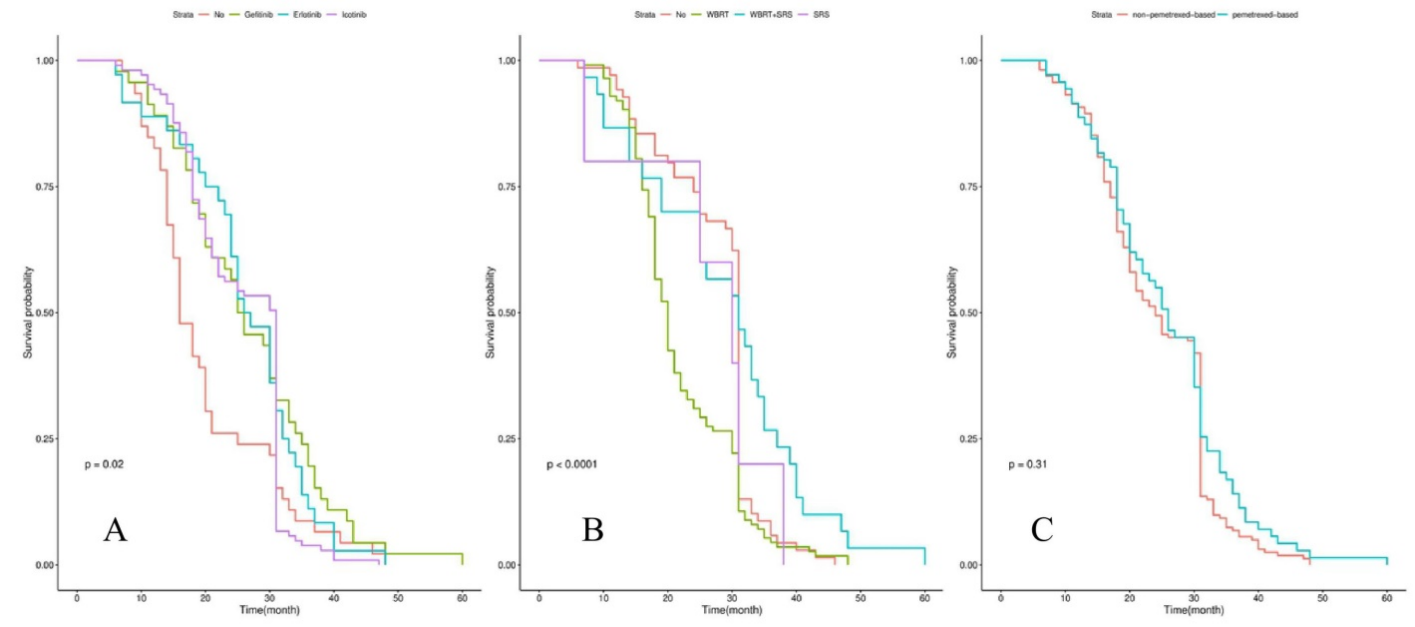
Kaplan-Meier curve analyses of OS in NSCLC patients with de novo brain metastases after EGFR-TKI, radiotherapy and chemotherapy. Kaplan-Meier curve was stratified by EGFR-TKI (gefitinib *vs* erlotinib* vs* icotinib) in A; radiotherapy (WBRT *vs* WBRT+SRS *vs* SRS) in B; chemotherapy (non-pemetrexed-based vs pemetrexed-based) in C. *Abbreviations: OS, overall survival; NSCLC: non-small cell lung cancer; EGFR-TKI, epidermal growth factor receptor-tyrosine kinase inhibitor; WBRT, whole brain radiation therapy; SRS, stereotactic radiosurgery.*

Survival analysis showed that the median iPFS and OS from diagnosis of BM were 12.0 (3.0-30.0) and 25.0 (6.0-60.0) months for the whole cohort. Patients with *EGFR* mutations had a significantly longer OS than those patients with *EGFR* wild-type (29.0 vs 16.0 months, P=0.004) in **Figure 1A**. The significant difference in OS was observed among groups of patients with* EGFR* 19 del, *EGFR* exon 21 L858R, and *EGFR* unknown mutations (30.5 vs 27.5 vs 16.0 vs 25.0 months, P=0.025) in **Figure 1B**. Patients treated by icotinib showed better OS than gefitinib and erlotinib (31.0 vs 25.5 vs 26.5, P=0.02) in **Figure 2A**. There was also a difference in OS of patients who received the whole-brain radiotherapy (WBRT), stereotactic radiosurgery (SRS), or WBRT+SRS (20.0 vs 31.0 vs 30.0 months, P<0.001), respectively in** Figure 2B**. No difference was found in the OS of patients treated by pemetrexed-based and non-pemetrexed-based chemotherapies (26.0 vs 24.0 months, P=0.31) in** Figure 2C**.

In the multivariate analysis, the prognosis of iPFS and OS in all advanced NSCLC patients with de novo BM were not correlated with the *EGFR* mutation subtype (HR=0.92 [95%CI (0.75-1.13)], P=0.428). Whilst those patients who received EGFR-TKIs treatment of icotinib had the better outcome of iPFS than gefitinib and erlotinib (HR=0.86 [95%CI (0.74-1.0)], P=0.04) but a trend in OS (HR=1.15[95%CI (0.99-1.33)], P=0.072). Also, the histology of non-adenocarcinomas, the number of BM (>3), and extracranial metastases status had independent negative impacts on the OS of all patients (P<0.001). All these data were shown in **Table 2**.

## Discussion

The prevalence of BM in NSCLC patients is increasing due to reliable imaging techniques, improved survival after novel regimens, the aging population, and advancing 9, 22. Although the systemic chemotherapy combined with local-control therapy is still the general discipline for advanced NSCLC patients with BM, compelling data has shown the superior efficacy of TKIs than chemotherapy as the first-line therapy in* EGFR* mutant patients 23-25. Based on those promising results, EGFR-TKIs have been recently recommended as the first-line therapy for never-smokers with adenocarcinoma of the lung having asymptomatic synchronous BM. However, the treatment of BM patients remains a big challenge for the unmet long-term survival goal. To our best knowledge, this is one of the largest real-world studies on advanced NSCLC patients with de novo BM across China focusing on the prognostic impact of the* EGFR* mutation and TKIs; as well as the clinicopathological characteristics and other therapeutic strategies. Firstly, the patients with different subtypes of *EGFR* mutations did not differ significantly in OS, but the discrepancy presented between *EGFR* mutant group and* EGFR* wild-type group. Secondly, the use of icotinib, one of the first-generation of EGFR-TKIs, was found to correlate with better iPFS of patients with *EGFR* mutation than other two commercial TKIs and a similar trend in OS. Finally, the histology of non-adenocarcinomas, diffuse BM (>3 intracranial lesions) and Extracranial metastases could have a negative independent impact on the OS in advanced NSCLC patients with de novo BM.

A high incidence of BM in NSCLC has been reported in* EGFR* mutation (40-60%) 26 or ALK alteration (about 50%) carriers 27. Moreover, the incidence of BM in patients with *EGFR* mutation is increasing during follow-up after curative surgery 28. The possible explanations for high-incidence of BM in oncogenic addiction NSCLC patients include the unique tumor characteristics and the low penetration rate of BBB of target therapy. Hsu et al. 29 reported a higher incidence of BM in* EGFR* mutation carriers than EGFR wild-type (39.2% *vs.* 28.2%, p=0.038) and significantly longer median survival (22.4 months *vs.* 7.9 months, p<0.001) in 534 patients with stage IV NSCLC. In our study, we confirmed the longer survival in* EGFR* mutant patients than* EGFR* wild-type which is consistent with the previous study. However, the *EGFR* mutation subtypes did not have an independent prognostic impact on de novo BM patients. Recently, Wang et al. 16 found the prognostic value of* EGFR* mutation in BMs and patients with the exon 19 deletion may have longer OS compared with exon 21 L858R mutation (not reached *vs* 26.5 months, P=0.0969). In the previous study in NSCLC with the de novo BM population, the extracranial metastatic foci and the response to treatment are important prognostic factors 7, 30. The patients enrolled in this study were mainly lung adenocarcinoma, females, non-smokers, and *EGFR* mutation carriers. It is not surprising that the histology, diffuse BM, and extracranial metastases could have an independent influence on the OS. Besides, more than half of our patients ever received WBRT as local treatment, and WBRT treated patients have the worst prognosis compared with SRS and a combination of WBRT+SRS. Although we are not trying to address the best timing of radiotherapy for de novo BMs, this finding indicates that the optimization of multimodality could weight more than regular local control for patients with BM 31.

Although the emerging promising efficacy of third-generation TKIs has been approved in advanced NSCLC with BM, the first generation of EGFR-TKIs are still commonly used as the first-line therapy for patients in China. We found that patients received icotinib had a longer median iPFS than patients treated by gefitinib and erlotinib. In a phase III trial (BRAIN), icotinib was associated with a significantly longer iPFS than WBRT combined with chemotherapy 32. Icotinib (BPI-2009H, Conmana) is an oral quinazoline compound that administered mainly in China for the lower expense, and it has a similar chemical structure to gefitinib and erlotinib 33. We assume that the superior of icotinib than other TKIs should be verified in further research since they have a similar mechanism of activity and therapeutic effects. The major challenge of TKIs in *EGFR* mutation patients with BM encountered is acquired drug resistance, which occurs very common in the first and second generation of TKIs 34. The new generation of TKIs including AZD3759 has been designed to overcome this problem 35. Regarding the efficacy in treatment and prophylaxis of BM by pemetrexed chemotherapy 36, we also compared the survival benefit of pemetrexed based or non-pemetrexed based chemotherapy in all patients including those with *EGFR* wild-type. No significant difference has been found in the survival analysis but further study is encouraged. Recently, PD-L1 positive NSCLC patients with BM could also benefit from immunotherapy 37. In summary, further research should continue finding the best therapy after the resistance of first-line TKIs in *EGFR* mutant BM patients and explore novel therapy for* EGFR* wild-type BM patients.

There are some limitations should be mentioned in our study. Firstly, we collected all the medical information retrospectively and the potential bias in patient selection could not be avoided. The involvement of surgical resection of primary and metastatic lesions has not been discussed in this study; Secondly, the baseline clinical characteristics of *EGFR* mutation patients in groups were not well balanced which could lead to the bias of conclusion. Third, the frequency of another important driver gene alteration* ALK* fusion should be presented due to its correlation with the development of BM in NSCLC patients 27. However, there were limited *ALK* detection data in our study and no ALK inhibitor has been administered either due to the lack of insurance coverage and approach for targeted therapy during that period. Finally, those treatments after first-line TKIs could likely influence the long-term survival of patients, as well as the documented adverse events and quality of life were lacking. Therefore, we were unable to analyze these potential prognostic factors in this study.

In summary, we discussed the prognostic influence of clinical characteristics and multimodality on advanced NSCLC patients with de novo BMs in a real-world study. Patients with different *EGFR* mutation subtypes may have a different response to TKIs which will turn out to have an impact on long-term survival. As the emerging results of novel target therapy and immunotherapy have shed light on the NSCLC patients with BM, larger prospective randomized clinical trials are urgently needed to explore the optimal multimodality in care for de novo BM patients as well for longer survival.

## Figures and Tables

**Table 1:** Characteristics of NSCLC patients with de novo brain metastasis in baseline (n=233).

Characteristic	Whole group, n=233 (100%)	EGFR 19del group, n=76 (32.6%)	EGFR 21L858R group, n=94 (40.4%)	EGFR wild-type group, n=46 (19.7%)	EGFR unknown group, n=17 (7.3%)	P value
Age						0.413
<65	189(81.1)	63(82.9)	73(77.7)	37(80.4)	16(94.1)	
≥65	44(18.9)	13(17.1)	21(22.3)	9(19.6)	1(5.9)	
Gender						0.971
male	60(25.8)	19(25)	25(26.6)	11(23.9)	5(29.4)	
female	173(74.2)	57(75)	69(73.4)	35(76.1)	12(70.6)	
ECOG PS						0.165
0-1	212(91)	68(89.5)	85(90.4)	44(95.7)	15(88.2)	
2-3	21(9)	8(10.5)	9(9.6)	2(4.3)	2(11.8)	
Smoking						<0.001
No	161(69.1)	60(78.9)	64(68.1)	21(45.7)	16(94.1)	
Yes	72(30.9)	16(21.1)	30(31.9)	25(54.3)	1(5.9)	
Histology						<0.001
*Non-adenocarcinoma	21(9)	0(0)	3(3.2)	18(39.1)	0(0)	
Adenocarcinoma	212(91)	76(100)	91(96.8)	28(60.9)	17(100)	
^#^Number of BM						0.644
Limited	120(51.5)	40(52.6)	44(46.8)	26(56.5)	10(58.8)	
Diffuse	113(48.5)	36(47.4)	50(53.2)	20(43.5)	7(41.2)	
Extracranial metastasis						0.531
No	99(42.5)	33(43.4)	37(39.4)	19(41.3)	10(58.8)	
Yes	134(57.5)	43(56.6)	57(60.6)	27(58.7)	7(41.2)	
^&^Radiotherapy						0.007
No	69(31.8)	27(38)	30(33.7)	11(26.2)	1(6.6)	
WBRT	113(52.1)	36(50.7)	50(56.2)	20(47.6)	7(46.7)	
SRS	30(13.8)	6(8.5)	7(7.9)	10(23.8)	7(46.7)	
WBRT+SRS	5(2.3)	2(2.8)	2(2.2)	1(2.4)	0(0)	
EGFR-TKIs						<0.001
No	46(19.7)	0(0)	0(0)	46(100)	0(0)	
Gefitinib	46(19.7)	14(18.4)	26(27.7)	0(0)	6(35.3)	
Erlotinib	36(15.5)	14(18.4)	16(17)	0(0)	6(35.3)	
Icotinib	105(45.1)	48(63.2)	52(55.3)	0(0)	5(29.4)	
Chemotherapy						0.453
Non-pemetrexed-based	162(69.5)	54(71.1)	69(73.4)	29(63)	10(58.8)	
Pemetrexed-based	71(30.5)	22(28.9)	25(26.6)	17(37)	7(41.2)	

^*^Non-adenocarcinoma included 3 cases with adeno-squamous carcinoma, 11 squamous carcinoma, 7 non-small cell lung cancer with unknown histologic subtype. ^#^Number of BM included the limited BM (1-3 intercranial lesions) and diffuse BM (>3 intercranial lesions). ^&^Sixteen patients were not having records of radiotherapy.

**Table 2:** Multivariate Cox regression analyses of iPFS and OS

Variable	iPFS			OS		
	P-value	HR	95%CI	P-value	HR	95%CI
EGFR Mutation	0.864	0.98	0.81-1.2	0.428	0.92	0.75-1.13
Smoking	0.422	1.14	0.83-1.57	0.718	1.06	0.77-1.46
Histology	0.032	0.54	0.31-0.95	<0.001	0.29	0.16-0.51
Number of BM	<0.001	1.97	1.47-2.66	<0.001	2.22	1.65-2.98
Extracranial metastases	0.001	1.67	1.24-2.24	<0.001	2.17	1.58-2.98
Radiation therapy	0.387	0.91	0.74-1.12	0.956	1	0.81-1.22
TKI	0.04	0.86	0.74-1.0	0.072	1.15	0.99-1.33
